# A primary amoebic meningoencephalitis case suspected to be infected by indoor swimming, China, 2024

**DOI:** 10.3389/fmed.2025.1623909

**Published:** 2025-09-03

**Authors:** Junfan Li, Songqi Feng, Yao Wang, Chao Li, Peng Li, Lijie Zhang, Yingxue Dai, Kaike Tan, Liang Wang

**Affiliations:** ^1^Department of Infectious Disease Control and Prevention, Chengdu Center for Disease Control and Prevention, Chengdu, China; ^2^Chinese Field Epidemiology Training Program, Chinese Center for Disease Control and Prevention, Beijing, China; ^3^Public Health Emergency Center, Chinese Center for Disease Control and Prevention, Beijing, China; ^4^Department of Endemic and Parasitic Disease, Chengdu Center for Disease Control and Prevention, Chengdu, China; ^5^Department of Environmental and School Health, Chengdu Center for Disease Control and Prevention, Chengdu, China

**Keywords:** primary amoebic meningoencephalitis, *Naegleria fowleri*, indoor swimming, field investigation, source of infection

## Abstract

**Introduction:**

*Naegleria fowleri* causes Primary Amoebic Meningoencephalitis (PAM), an unusual but fatal disease. Swimming in wild freshwater is commonly regarded as the primary cause of infection. In April, 2024, we discovered a case of PAM in a child, suspected to have contracted the infection through an uncommon route: indoor swimming. This article describes the field epidemiological investigation process, the reasoning behind the speculation of the infection origin, and recommendations for minimizing potential risks.

**Methods:**

Face-to-face visit was conducted with the guardian of the case. Field investigations were carried out at suspected venues of infection. Reasonable inferences were made by combining literature and investigation results.

**Results:**

The case manifested typical PAM symptoms, followed by death 7 days after onset. The suspected exposure period was between March 4 and April 3, associated with several instances of swimming in two indoor pools. A field investigation at N Aquatic Center revealed improper disinfection methods, substandard water quality, and a suitable environment for *Naegleria fowleri*.

**Discussion:**

N Aquatic Center is the most likely source of infection after synthesizing epidemiological history, field investigation, and laboratory results. Individuals can get infected even when swimming indoors if the water is not well managed. Inspecting, monitoring, and disinfecting pool water should be strengthened.

## 1 Introduction

Primary amoebic meningoencephalitis (PAM) is a rare but fatal condition caused by *Naegleria fowleri*, with a fatality rate reaching as high as 95% (1). The early symptoms of the disease are non-specific, mostly presenting flu-like symptoms, including fever, headache, and vomiting (2–4). However, the disease progresses rapidly, leading to severe central nervous system damage within days of the initial symptoms (5). Additionally, the process of diagnosing is challenging, typically involving the microscopic examination of cerebrospinal fluid (CSF) (6), polymerase chain reaction (PCR) (7), and sequencing (8) when facing unknown meningoencephalitis. Microscopic examination of CSF lacks sensitivity, and the results are difficult to distinguish from other bacterial meningoencephalitis (9). More sensitive technology, such as metagenome next-generation sequencing (mNGS) can identify several underlying pathogens, which can be decisive in the diagnosis of unknown diseases. However, mNGS is more expensive and less accessible, limiting its application in the early stages of diseases.

Worldwide, *Naegleria fowleri* is prevalent, particularly in warmer regions (10). The earliest probable case of PAM dates back to 1961 in Australia (11), and as of 2021, a total of 381 cases has been reported from 33 countries on most continents (12). Researchers identify contaminated water as the most common route of infection (13, 14). Thus, in most PAM cases, individuals have histories of engaging in water activities, including swimming or diving, particularly in wild freshwater (15). However, *Naegleria fowleri* cannot easily infect individuals by simply touching, using, or playing with water, risk only rises when the pathogen reaches nasal cavity (16). The pathogen can attach itself to the nasal mucosa, then penetrate it and migrate along olfactory nerves through the cuneiform plate, and finally reaches the brain, where the pathogen multiplies and causes severe neurological damage (17–19). Therefore, nasal irrigations could also be risky if the water was contaminated (20).

On April 9, 2024, The J district CDC of Chengdu city reported that a 6-year-old child was diagnosed with PAM. The *Naegleria fowleri* test via mNGS confirmed positive results. A collaborative investigation between municipal and district CDCs was conducted to trace the possible source of infection, assess the risk to the population, and provide recommendations to prevent further hazards.

## 2 Materials and methods

### 2.1 Epidemiologic investigation

Face-to-face visit was conducted with the guardian of the case. In addition to routine elements of the epidemiological investigation, the visits focused on suspected contaminated-water-exposing behaviors like swimming, diving, nasal irrigation, or other water activities, to trace the origin of the infection.

### 2.2 Field hygiene investigation

Field investigations on hygiene were conducted at suspected sources of infection. The field investigation began with an inspection of the venue’s infrastructure to determine if equipment was adequate and well-maintained. Another aspect of the field investigation was related to disinfection, consisting of disinfection methods, disinfection frequency, disinfection records, and evaluation of the disinfection effects of pool water. On-site water tests were also performed, and the results were determined with reference to the national standard document *Hygienic indicators and limits for public places* (GB 37488-2019).

### 2.3 Environment and case sampling

Water samples were collected with reference to the national standard document *Examination methods for public places-Part 6: Technical specifications of health monitoring* (GB/T 18204.6), and the sampling points and volume were increased on the request of the national standard to improve the probability of detecting pathogens. For microbiological testing, sampling points were selected at four corners and center of the waters. Sterilized containers were used for sampling, where 1 mL of 10% sodium thiosulfate in advance had been added to neutralize residual disinfectant in the water samples, and 1000 mL of water was collected 30 cm below the water surface. All samples were transported immediately to the laboratory under refrigerated conditions.

To obtain case’s CSF, the child was placed in the left lateral position, with the neck flexed and the hip flexed. The lumbar 3-4 vertebral space was used as the puncture point. Prior to the procedure, the surgical site was meticulously disinfected, and sterile gloves were donned. Sterile towels were then spread on the operating table. The lumbar puncture needle was inserted vertically into the patient’s back along the puncture point. Two breakthrough sensations were felt after penetrating the skin, prompting the injection to be halted. The needle core was then extracted to observe the egress of light, bloody, turbid CSF at a rate of approximately 10 drops per minute. A total of 3 m1 of this fluid was subsequently sent for analysis, including routine CSF testing, biochemistry testing, and mNGS to the reinsertion of the needle core and the extraction of the puncture needle, the iodophor was sterilized, covered with sterile gauze, and secured with adhesive tape. The operation proceeded without incident, and following the lumbar puncture, the patient was stripped of pillows and was positioned in a recumbent position for a period of 6 h.

### 2.4 Laboratory tests

Microbiological, physical and chemical testing were performed on the waters to reveal the water quality. Total colony count, total coliforms and other pathogenic bacteria indicated microbial activities of the water body. Free residual chlorine revealed status of disinfectants using, substandard suggested improper use of disinfectants and risks due to failure to kill pathogens. Water temperature, pH, turbidity, redox potential reflected the physical and chemical environment of the water body, poor environment contributed to the survival of pathogens.

Tests of CSF, cranial computed tomography (CT) and mNGS were performed on the case to identify the pathogen of disease. Biochemical and microscopic examination of CSF evaluated the status of central nervous system health, assisted in the diagnosis of disease, and helped search for microscopic pathogens. CT displayed the structure and morphology of the brain in different cross-sections, helping to determine the extent and severity of infection. mNGS sequenced the DNA in the samples and analyzed the microorganisms in the samples through sequence comparison with known microbial genomes in the database based on high-throughput sequencing technology. The detection process included the following steps: nucleic acid extraction, library construction, sequence determination, data analysis and information interpretation. The scope of detection includes known microorganisms such as bacteria, archaea, mycoplasma, chlamydia, nuclease, spirochetes, viruses and fungi.

## 3 Results

### 3.1 Main clinic symptoms and key time points

**FIGURE 1:**
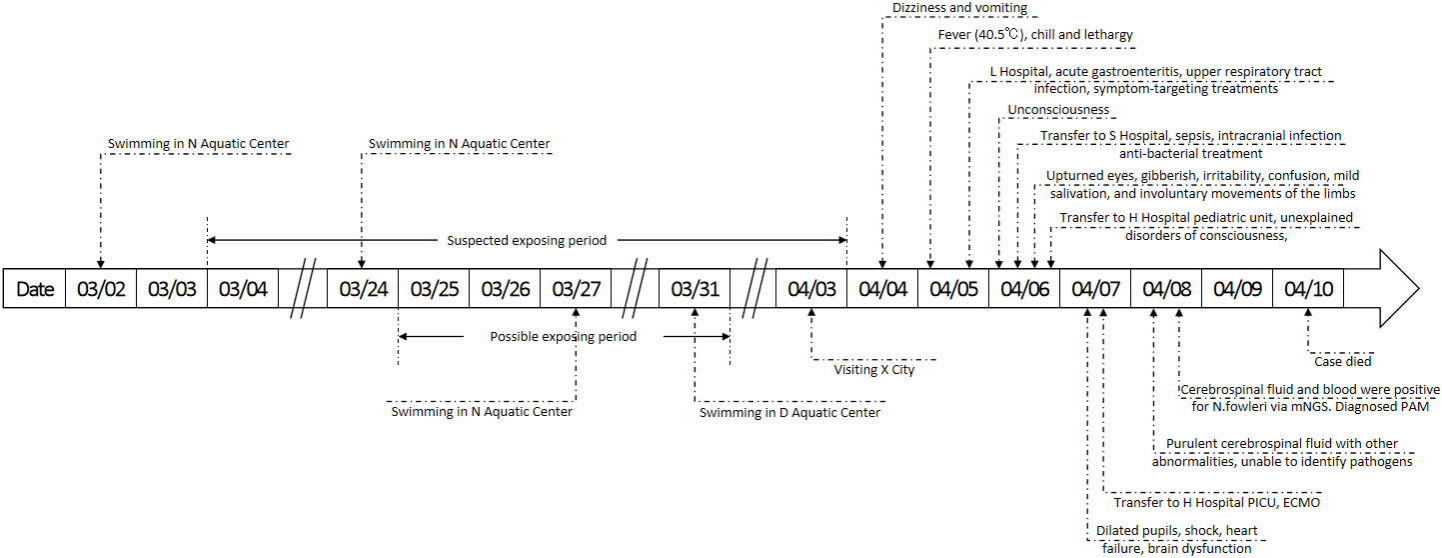
Details of the timeline, symptoms, diagnosis and treatments. mNGS, metagenome next generation sequencing; PAM, primary amoebic meningoencephalitis; PICU, pediatric intensive care unit; ECMO, extracorporeal membrane oxygenation.

The case was a 6-year-old child with a previously healthy condition. Symptoms began on April 4 in Xichang City of Sichuan Province, starting with dizziness at noon and vomiting once in the afternoon. Fever appeared (peak 40.5 °C) in the early morning of April 5, accompanied by chills and lethargy. The case took ibuprofen twice, but it did not reduce the fever. The case sought care at L Hospital in Xichang City and received a diagnosis of “acute gastroenteritis and upper respiratory tract infection.” L Hospital provided treatments targeting symptoms, but they were not effective. On April 6, the case left Xichang City for Chengdu City for better care but developed unconsciousness during the transfer. The case was admitted to S Hospital in Chengdu City of Sichuan Province the same afternoon with the main diagnosis of “sepsis and intracranial infection.” S Hospital provided antibacterial treatment alongside symptom-targeting treatments. At 18:00 on April 6, the case suddenly developed more neurological symptoms, including upturned eyes, gibberish, irritability, confusion, mild salivation, and involuntary movements of the limbs. The physician’s physical assessments revealed dilated pupils and a diminished light response. At 21:00, the pediatric unit at H Hospital admitted the case with the main diagnosis of “unexplained disorders of consciousness.” In the early morning of April 7, the case’s condition rapidly progressed, manifesting dilated pupils, shock, coma, heart failure, and brain dysfunction. The case was promptly admitted to the Pediatric Intensive Care Unit (PICU) with a diagnosis of shock, heart failure, and brain dysfunction. Extra Corporeal Membrane Oxygenation (ECMO) was applied for rescue. On April 10, the case died. The details of the symptoms, diagnosis, and treatments are shown in Figure 1.

### 3.2 Case laboratory results

**FIGURE 2:**
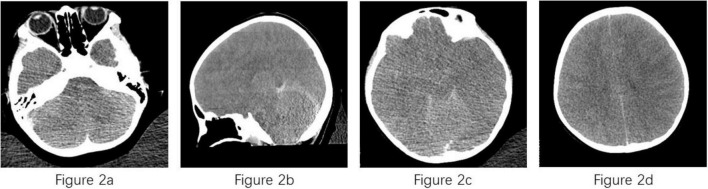
Computed tomography of case. **(a)** Diffuse swelling on both sides of brain; **(b)** unclear outline of the brainstem; **(c,d)** loss of brain sulcus, cistern and ventricles.

Examinations of the CSF on April 8 revealed a purulent specimen with abnormalities in several biochemical indices, including glucose (4.76 mmol/L), lactate dehydrogenase (2845 U/L), and CSF proteins (>10000 mg/L), however CSF specimen volume too low to perform cell counts. Peripheral blood analysis showed elevated white blood cell count (20700 cells/μL) with a neutrophilic predominance. CT displayed diffuse swelling on both sides of the brain, unclear outline of the brainstem, loss of brain sulcus and cistern, narrowing and loss of the third and fourth ventricles, and brain herniation was considered to have formed (Figure 2). However, the underlying pathogen remained unidentified. After applying mNGS, the CSF and blood both tested positive for *Naegleria fowleri*, with 10314 bp of reads of microbial detection sequences and 99.94% of relative abundance for CSF, 2503 bp of reads of microbial detection sequences and 99.90% of relative abundance for blood, respectively, leading to the diagnosis of PAM in the case.

### 3.3 Epidemiologic history and speculation of the exposure period

The case had not been swimming, diving, or engaging in other similar water activities in any wild freshwater for the past 3 months but had consistently engaged in regular swimming in indoor pools since the age of one. The case frequented N Aquatic Center on March 3, March 24, and March 27, and D Aquatic Center on March 31. Other suspected contaminated-water exposure was visiting Xichang City, famous for its lake view, on April 3. However, this exposure was ruled out as the case only stayed near the lake without any behavior that could have led to nasal water ingress. The possibility of being infected at home was also ruled out, as routine household water behaviors rarely involved submerging nasal cavity, the epidemiologic history again did not reveal the case with a habit of nasal irrigation. Details of the epidemiologic history are shown in Figure 2.

Given the characteristics of the pathogen and the disease pathophysiology, the hypothesis was made that contacting with *Naegleria fowleri*-contaminated water was the cause of infection. As the median incubation period of PAM is about 6 days (12), our initial speculation suggested that the case was most likely exposed between March 25 and March 31, approximately 1 week prior to the onset of symptoms. To guarantee the accomplishment of tracing the origin of infection and given that the possible incubation period could range from 1 to 30 days (12), the final speculation of the exposing period was prolonged between March 4 and April 3. After cross-comparing the hypothesis and suspected water-contamination behaviors, swimming in the N Aquatic Center on March 24 and 27 and swimming in the D Aquatic Center on March 31 were identified as potential causes. Then, field hygiene investigations were conducted at both venues.

### 3.4 Field hygiene investigation at the N Aquatic Center

The N Aquatic Center, situated in H District of Chengdu City, has been operating a single pool since 2019. The length, width, and depth of the pool were 18 m, 8 m, and 1.1 m, respectively. The interior tile-covered surface of the pool had several cracks or breaks, and it lacked a foot-sanitizing sink. The water was heated to a temperature of 32 °C. Approximately one-third of the water was replaced weekly, and the pool was recently emptied and cleaned on February 22, 2024. Methods for disinfection included the use of ultraviolet (UV) light and mechanical filtration. However, the operating status of the UV lights was unknown, and the filter could only block hair. Additional ozone disinfection was applied from 21:00 to 8:00 each night, but no chlorine disinfectant was used at any time. The venue lacked a daily water quality monitoring program and corresponding records. An independent company prepared the most recent testing report on July 15, 2023, which revealed an overall result of passing. On-site testing for pool water was performed on April 10, 2024, the day of investigation. The results did not meet the requirements for pool water according to the national standard document (GB 37488-2019). The free residual chlorine was 0.00 mg/L (0.30–1.00 mg/L), the total bacterial count was 220 CFU/mL (≤200 CFU/mL), and the coliform group was 3 CFU/100 mL (not allowed). *Pseudomonas aeruginosa* was also detected but not for *Naegleria fowleri*.

### 3.5 Field hygiene investigation at the D Aquatic Center

D Aquatic Center, located in L District of Chengdu City, is a newly built, large-scale, advanced venue with a capacity of 5,000 people. It hosted international water sports events in 2023. The venue consists of three pools: one open to the public, one for competition, and one for diving only. The case participated in a swimming competition on March 31 and only came into contact with water in the competition pool. The competition pool was 50 m in length, 25 m in width, and 2 m in depth. The pool’s interior tile-covered surface was clean and intact. The water temperature was maintained between 26 °C and 28 °C, and about 5% of the water was replaced daily. An integrated system managed the water and could display real-time monitoring results, including free residual chlorine, potential of hydrogen (pH), temperature, turbidity, and oxidation-reduction potential (ORP). All indicators were in accordance with standards on the day we investigated, April 10, 2024. The venue also established a daily manual sampling and testing mechanism. Records and corresponding photographs of manual sampling on March 31, 2024, the day the case swam, were carefully reviewed, revealing satisfactory results of water quality. Disinfection methods at the venue were diatomite filtration, ozone, and sodium hypochlorite. The system automatically managed and applied all methods based on real-time monitoring results. On-site inspection of relevant equipment revealed satisfactory operating status. On-site testing for pool water was also performed during the investigation. The results were completely in compliance with the standard document (GB 37488-2019). The free residual chlorine was 0.84 mg/L (0.30–1.00 mg/L), the total bacterial count was 110 CFU/mL (≤200 CFU/mL), and the coliform group was not detected (not allowed). Other pathogens, including *Naegleria fowleri*, were not detected. Comparison of hygiene investigation results between two aquatic centers was shown in Table 1.

**TABLE 1:** Comparison of hygiene investigation results between N and D Aquatic Center.

Contents	N Aquatic Center	D Aquatic Center	Requirements of GB 37488-2019
Location	H District	L District	N/A
Year of opening	2019	2023	N/A
Scale	Small pool, 18 m × 8 m × 1.1 m	A newly built, large-scale, international-sport- ready aquatic center, composed of two 50 m × 25 m × 2 m swimming pools and one diving pool.	N/A
Foot-bath sink	None	Equipped	N/A
Maintenance	Poor. Interior tile was detached in 3 places with dark spots of unidentified origin	Good. All tile-covered surface was clean and intact	N/A
Water replacement	33% per week	5% per day	N/A
Disinfection methods	Mechanical filtration, ozone and ultraviolet (UV)	Diatomite filtration, ozone, sodium hypochlorite and etc.	N/A
Evaluation of disinfection effect	Absence of real-time monitoring. Most recent testing report issued on July 15, 2023	Real-time monitoring of water quality including free residual chlorine, potential of hydrogen (pH), temperature, turbidity, and oxidation-reduction potential (ORP). Daily manual sampling and tests for water quality with traceable records	N/A
Disinfection period	UV: throughout the day: Ozone: 21:00 – 8:00 every night	Automatic dosing, water replacement, and other water maintenance based on real-time monitoring	N/A
Water temperature	32 °C	26 °C	23 °C–30 °C
Free residual chlorine	0.00 mg/L	0.84 mg/L	0.30–1.00 mg/L
Total bacterial count	220 CFU/mL	110 CFU/mL	≤200 CFU/mL
Coliform group	3 CFU/100 mL	0 CFU/100 mL	0 CFU/100 mL
Other pathogen	Pseudomonas aeruginosa was detected	None	N/A

## 4 Discussion

The case reported in this study was the first case of PAM in Chengdu city, the clinic symptoms, laboratory testing results and epidemiologic history shared similarities with previous cases reported worldwide. The early symptoms in our case were non-specific, including dizziness, vomiting and fever, which has been reported in other cases before (21, 22). Other early and overlooked symptoms included headache (23) and nausea (24), making it hard to pay enough attention to PAM at an early stage. It was also extremely difficult to diagnose PAM even after patients have sought medical attention, our case was merely diagnosed as respiratory and gastroenteric diseases before the neurological damage was present; Although CSF tests revealed obvious abnormalities, the diseases suggested by indicators was indistinguishable from bacterial meningitis (25). All cases had a history of suspected contaminated-water exposure due to the route of infection, but few was associated with artificial water. However, US once reported a PAM case due to exposing to inadequately chlorinated recreational waters (26), with almost identical epidemiologic history to ours, suggesting that focusing our investigation on aquatic centers was proper.

The field investigation conducted at the two aquatic centers provided evidence to reasonably deduce that *Naegleria fowleri* did survive in the water of the N Aquatic Center, although failing to detect the pathogen. The results at N Aquatic Center revealed that the water quality was substandard, the infrastructure was outdated and damaged, the water monitoring and management mechanisms were absent, and the disinfection methods were inadequate. The on-site investigation revealed multiple cracks and breaks on the interior surface of the pool. Unlike the intact tile-covered portion, the damaged areas were rougher, with dark spots of unidentified origin, likely indicative of microbial activity. A previous study (27), reported the isolation of *Naegleria fowleri* from damaged pool walls, which lends support to our investigation and speculation. Additionally, the inadequate infrastructure, including the lack of a foot-sanitizing sink, increased the probability that *Naegleria fowleri* was carried into the pool from outside. Moreover, the microbial indices of water at the N Aquatic Center significantly exceeded limits, and *Pseudomonas aeruginosa* was detected, indicating a variety of highly active microorganisms in the water. Consequently, the pool water can be considered wild freshwater to some extent, providing suitable conditions for the survival of *Naegleria fowleri*. The relatively high water temperature of 32 °C was also suitable for *Naegleria fowleri*, as it is commonly found in warm environments, both artificial and natural (28). Lastly, the disinfection methods may not have been effective enough to eliminate *Naegleria fowleri*. Some literature reports that chlorine is critical to controlling the spread of *Naegleria fowleri* (29, 30), and even free residual chlorine levels as high as 1.5 mg/L may not adequately eliminate the pathogen (31), and the combination of the UV light with the chlorination allowed the complete removal of the *Naegleria fowleri* trophozoites from the water (32). However, the venue only applied UV and ozone disinfection, and the on-site water testing results showed 0 mg/L of free residual chlorine. The detailed operating status of UV disinfection was not available to us during the investigation. The duration, intensity, and other indicators of UV may have fallen short of meeting the disinfection requirements for the water body. During the investigation, the ozone disinfection system was also not operating, as it was scheduled to be applied at midnight. Not to mention that neither records of disinfection nor periodic evaluations of disinfection were available. It was indeed hard to believe that the disinfection methods implemented at the N Aquatic Center were working as they should.

There were also some limitations in this study. We did not find *Naegleria fowleri* in the pool water of N Aquatic Center, although findings of epidemiologic investigation and field hygiene investigation both pointed to the pool as the source of infection, direct evidence of existence of pathogen was absent. We did not test the source water of the pool due to the accessibility of investigations, therefore it could not be determined that whether the source of pathogen was due to contamination of tap water or irregularities in the disinfection of the pool.

After synthesizing the epidemiological history, field investigation, and laboratory results, the most likely source of infection could be swimming in the N Aquatic Center. Although the suspected contaminated-water-exposure history was complicated, only activities such as swimming, which involve filling the nose with water, gave *Naegleria fowleri* the opportunity to attach to the nasal mucosa and cause infection. Based on the summarized information and the epidemiological history, including the onset time of symptoms and incubation period, only three swimming experiences at two sites were identified as suspicious. Taking into account the results of the field investigation at both locations, N Aquatic Center was the most probable origin of the infection.

## 5 Conclusion

The case was most likely infected through indoor swimming based on the information synthesized. According to the findings of the investigation, water that should have been clean became a vehicle for infection. Thus, there is an urgent need to strengthen the supervision of disinfecting in certain venues to prevent potential future infections. Additionally, based on the pathogen characteristics of *Naegleria fowleri*, it is recommended to avoid swimming in wild water.

## Data Availability

The original contributions presented in this study are included in this article/supplementary material, further inquiries can be directed to the corresponding author.
